# Short- and Long-Term Outcomes in Patients With Right Ventricular Infarction According to Modalities of Reperfusion Strategies in China: Data From China Acute Myocardial Infarction Registry

**DOI:** 10.3389/fcvm.2022.741110

**Published:** 2022-02-10

**Authors:** Mengjin Hu, Ge Chen, Hongmei Yang, Xiaojin Gao, Jingang Yang, Haiyan Xu, Yuan Wu, Lei Song, Shubin Qiao, Fenghuan Hu, Yang Wang, Wei Li, Chen Jin, Yuejin Yang, for the China Acute Myocardial Infarction Registry Investigators

**Affiliations:** ^1^State Key Laboratory of Cardiovascular Disease, National Center for Cardiovascular Diseases, Fuwai Hospital, Chinese Academy of Medical Sciences & Peking Union Medical College, Beijing, China; ^2^First Hospital of Qinhuangdao, Qinhuangdao, China

**Keywords:** right ventricular infarction, primary PCI, reperfusion strategy, thrombolysis, myocardial infarction

## Abstract

**Purpose:**

We sought to investigate the short- and long-term outcomes in patients with right ventricular infarction in China.

**Methods:**

Data from China Acute Myocardial Infarction (CAMI) Registry for patients with right ventricular infarction between January 2013 and September 2014 were analyzed.

**Results:**

Of the 1,988 patients with right ventricular infarction, 733 patients did not receive reperfusion therapy, 281 patients received thrombolysis therapy, and 974 patients underwent primary PCI. Primary PCI and thrombolysis were all associated with lower risks of in-hospital (3.1 vs. 12.6%; adjusted OR: 0.48; 95% CI: 0.27–0.87; *P* = 0.0151 and 5.7 vs. 12.6%; adjusted OR: 0.43; 95% CI: 0.22–0.85; *P* = 0.0155, respectively), and 2-year all-cause mortality (6.3 vs. 20.9%; adjusted HR: 0.50; 95% CI: 0.34–0.73; *P* = 0.0003 and 11.0 vs. 20.9%; adjusted HR: 0.59; 95% CI: 0.38–0.92; *P* = 0.0189, respectively), compared with no reperfusion therapy. Meanwhile, primary PCI was superior to thrombolysis in reducing the risks of in-hospital atrial-ventricular block (4.2 vs. 8.9%; adjusted OR: 0.46; 95% CI: 0.23–0.91; *P* = 0.0257), cardiogenic shock (5.3 vs. 13.9%; adjusted OR: 0.43; 95% CI: 0.23–0.83; *P* = 0.0115), and heart failure (8.5 vs. 23.5%; adjusted OR: 0.35; 95% CI: 0.22–0.56; *P* < 0.0001). Primary PCI could reduce the risk of 2-year major adverse cardiac and cerebrovascular event (19.1 vs. 33.3%; adjusted HR: 0.72; 95% CI: 0.56–0.92; *P* = 0.0092) relative to no reperfusion therapy, whereas thrombolysis may increase the risk of 2-year revascularization (15.5 vs. 8.7%; adjusted HR: 1.90; 95% CI: 1.15–3.16; *P* = 0.0124) compared with no reperfusion therapy.

**Conclusions:**

Timely reperfusion therapy is essential for patients with right ventricular infarction. Primary PCI may be considered as the default treatment strategy for patients with right ventricular infarction in the contemporary primary PCI era.

## Introduction

Right ventricular infarction occurs in a substantial proportion of patients with acute inferior myocardial infarction and is associated with increased rates of morbidity and mortality (1). However, in contrast to the effects of coronary occlusion on regional and global left ventricular function (2), early studies suggested that even in the absence of reperfusion of the infarct-related artery (IRA), most patients with severe ischemic right ventricular dysfunction manifest spontaneous early hemodynamic improvement and later recovery of right ventricular function (3, 4). Although the salutary effects of timely reperfusion have been well-documented, yet the population is based on patients with left ventricular infarction (5), and the strategy to treating right ventricular infarction has not been adequately evaluated and remains little controversial. Some studies suggested that right ventricular function was recovered only after successful reperfusion (4, 6, 7), whereas others reported improvement even in the absence of a patent IRA (8). Moreover, these studies were performed before the primary percutaneous coronary intervention (PCI) era, and contemporary primary PCI practice and medical management have evolved and are significantly different from the earlier one. Therefore, we performed a study designed to investigate the current prevalence, short- and long-term outcomes in Chinese patients with right ventricular infarction in the contemporary primary PCI era, using a large database representing real-world Chinese patients with right ventricular infarction.

## Methods

### Study Population

Details of the China Acute Myocardial Infarction (CAMI) Registry have been previously described (9). Briefly, the CAMI Registry is a prospective, nationwide, multicenter observational study enrolling AMI patients between January 2013 and September 2014. A total of 108 hospitals in 27 provinces and 4 municipalities in Mainland China participated, including 31 provincial hospitals, 45 municipal hospitals in their own provinces or municipalities, and 32 county hospitals in these selected prefectures, with a broad coverage of geographical regions, including urban and rural areas. Written informed consent was obtained from eligible patients before registration, and the study protocol conforms to the ethical guidelines of the 1975 Declaration of Helsinki. This study was approved by our institutional review board committee and registered on www.clinicaltrials.gov (NCT01874691). Data were collected, validated, and submitted through a secure, web-based electronic data capture system. Follow-up was carried out by trained physicians at each participating site in a real-time manner to ensure data accuracy and reliability. Senior cardiologists were responsible for the data quality control and periodic database checking was undertaken (9). AMI is diagnosed according to the third universal definition of myocardial infarction (10). The clinical triad of hypotension, clear lung fields, and an elevated jugular venous pressure has been traditionally considered a marker of right ventricular infarction. On a right-sided electrocardiography, ST-segment elevation of more than 1 mm in lead V4R is considered significant. Indirect signs of right ventricular infarction in echocardiogram includes right ventricular dilation, tricuspid regurgitation, reduced excursion of the tricuspid annulus, and dilation of the inferior vena cavae (11, 12). Right ventricular infarction was diagnosed according to the aforementioned clinical symptoms, electrocardiography, and echocardiogram results. Major bleeding was defined according to the Thrombolysis in Myocardial Infarction (TIMI) classification (13). In the present analysis, STEMI patients with right ventricular infarction were included.

### Clinical Outcomes

The primary outcome was all-cause mortality. The secondary outcome was major adverse cardiac and cerebrovascular event (MACCE), which was defined as a composite of all-cause mortality, AMI, revascularization (PCI/coronary artery bypass graft), stroke, and major bleeding. Meanwhile, the outcomes of ventricular flutter/fibrillation, atrial flutter/fibrillation, sinus arrest/severe bradycardia, atrial-ventricular block, cardiogenic shock, and heart failure during admission were also investigated.

### Statistics

Continuous normally distributed variables were expressed as means ± standard deviation (SD) and compared using the Student's unpaired *t*-test. Continuous non-normally distributed variables were expressed as median and interquartile ranges and analyzed using the Mann–Whitney *U*-test. Categorical variables were expressed as numbers and percentages, and compared with the χ^2^-test when applicable; otherwise, Fisher's exact test was adopted. Cumulative incidences of clinical events were estimated using the Kaplan–Meier method, and differences were evaluated with the log-rank test. Multivariable Cox proportional-hazards models were used to assess the risk of thrombolysis and primary PCI relative to no reperfusion therapy for the primary, secondary, and other outcomes, expressed as hazard ratios (HRs) and its 95% confidence interval (CI). The adjusted variables included age, sex, symptoms onset to admission time, smoking, heart failure, previous MI, hypertension, hyperlipidemia, diabetes, stroke, Killip class, cardiogenic shock, left ventricular ejection fraction (LVEF), angiotensin-converting enzyme inhibitor/angiotensin receptor blocker (ACEI/ARB), β receptor blocker, GRACE risk score, and hospital level. All statistical tests were two-sided with a 5% level of significance, and the analyses were performed using SAS 9.4 software (SAS Institute, Cary, North Carolina).

## Results

**Figure 1:**
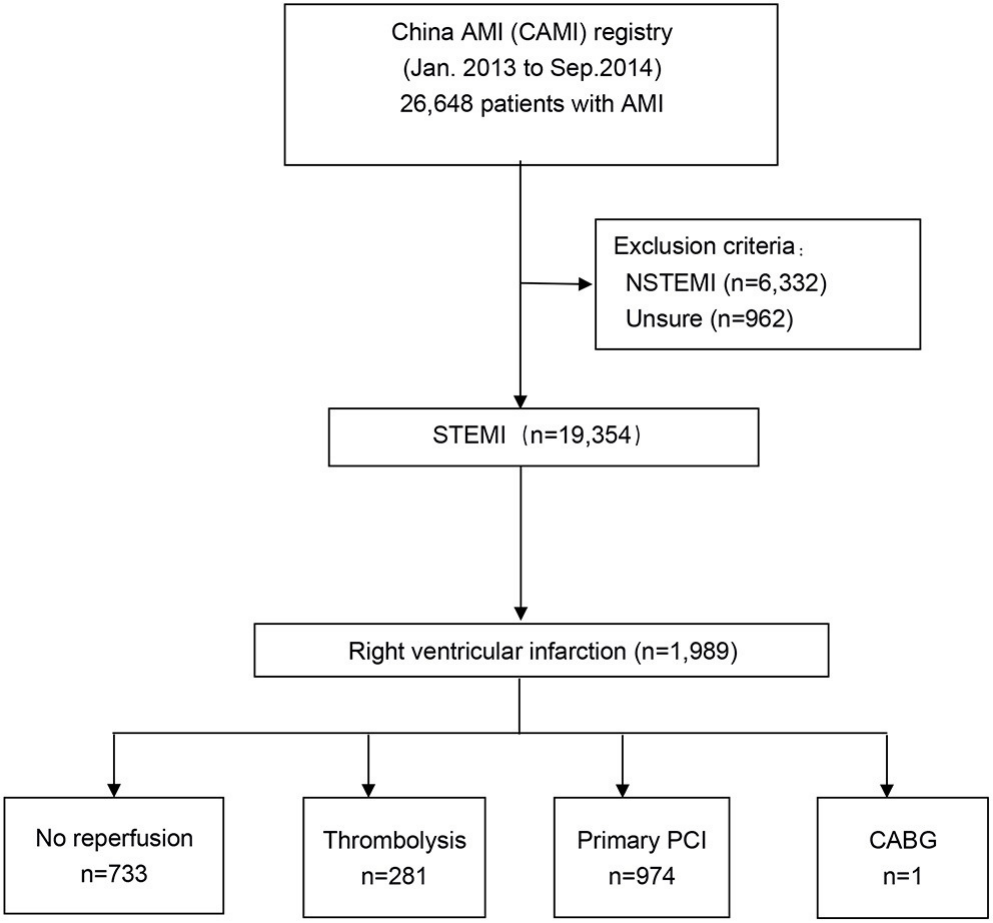
Study flow. The study population was derived from the nationwide, multicenter, prospective China Acute Myocardial Infarction (CAMI) Registry. CABG, coronary artery bypass graft; NSTEMI, non–ST-segment elevation myocardial infarction; PCI, percutaneous coronary intervention; STEMI, ST-segment elevation myocardial infarction.

A total of 26,648 patients with AMI were included in our CAMI registry from January 2013 to September 2014. We excluded patients with non-ST-elevation myocardial infarction (NSTEMI; *n* = 6,332) or unconfirmed information (*n* = 962). Eventually, a total of 1,988 patients with STEMI associated with right ventricular infarction were included in our study. We categorized these patients into three groups according to treatment strategy: no reperfusion group (*n* = 733; 36.9%), thrombolysis group (*n* = 281; 14.1%), and primary PCI group (*n* = 974; 49.0%). The study flow chart is shown in Figure 1.

The comparison of patient characteristics among no reperfusion, thrombolysis, and primary PCI groups is shown in Table 1. Patients treated with no reperfusions were oldest and most likely to be diabetic patients. They spent the longest time from symptom onset to medical care, in the intensive care unit (CCU), and in hospital admission. Moreover, the GRACE risk score was also highest in the no reperfusion group. However, they were least likely to be smokers and used the least aspirin, clopidogrel/ticagrelor, or statins. Patients in the thrombolysis group were most likely to manifest cardiogenic shock, arrhythmia, and cardiac arrest. Defibrillation, morphia, and heparin were used most in the thrombolysis group. Meanwhile, after hospital discharge, patients in the thrombolysis group used most aspirin, clopidogrel/ticagrelor, nitrate, and statins. On the other hand, patients treated with primary PCI were most likely to be current smokers and hyperlipidemia patients. The level of left ventricular end-diastolic dimension (LVEDD) was lowest, whereas LVEF was highest in primary PCI group. They were most likely to use temporary pacemakers, intra-aortic balloon pump (IABP), GP IIb/IIIa receptor antagonists, and β receptor blockers. No reperfusion and thrombolysis therapies were most likely to be seen in municipal hospitals, whereas primary PCI practice was most prevalent in provincial hospitals.

**Table 1:** Baseline characteristics according to treatment strategies in patients with right ventricular infarction.

	**No reperfusion (*n* = 733)**	**Thrombolysis (*n* = 281)**	**Primary PCI (*n* = 974)**	***P*-value**
**Demographic characteristics**				
Age	64.05 ± 12.74	60.57 ± 11.46	60.57 ± 11.46	<0.0001
Age ≥ 75 ys	161 (22.0)	27 (9.6)	107 (11.0)	<0.0001
Male	526 (71.8)	219 (77.9)	736 (75.6)	0.0735
BMI	24.02 ± 3.20	24.58 ± 3.03	25.04 ± 13.38	0.0969
**Risk factors**				
Smoker	405 (55.3)	172 (61.2)	594 (61.0)	0.0412
Current smoker	332 (45.3)	138 (49.1)	515 (52.9)	0.0080
Hypertension	356 (48.6)	129 (45.9)	503 (51.6)	0.1768
Diabetes	159 (21.7)	40 (14.2)	186 (19.1)	0.0221
Hyperlipidemia	46 (6.3)	17 (6.0)	102 (10.5)	0.0025
Prior MI	48 (6.5)	25 (8.9)	58 (6.0)	0.2388
Prior PCI	31 (4.2)	12 (4.3)	44 (4.5)	0.9551
Prior CABG	4 (0.5)	0 (0.0)	3 (0.3)	0.2581
Heart failure	17 (2.3)	4 (1.4)	9 (0.9)	0.0673
Stroke	85 (11.6)	32 (11.4)	81 (8.3)	0.0552
Peripheral vascular diseases	4 (0.5)	0 (0.0)	8 (0.8)	0.1264
Renal failure	8 (1.1)	2 (0.7)	11 (1.1)	0.8115
COPD	16 (2.2)	2 (0.7)	11 (1.1)	0.1074
**Hospital level**				<0.0001
Provincial level	306 (41.7)	40 (14.2)	554 (56.9)	
Municipal level	330 (45.0)	142 (50.5)	358 (36.8)	
County level	97 (13.2)	99 (35.2)	62 (6.4)	
**Symptoms onset to admission time**				<0.0001
<3 h	114 (15.6)	154 (54.8)	359 (36.9)	
3–6 h	130 (17.7)	98 (34.9)	369 (37.9)	
7–12 h	99 (13.5)	21 (7.5)	177 (18.2)	
13–24 h	136 (18.6)	3 (1.1)	42 (4.3)	
2–7 d	254 (34.7)	5 (1.8)	27 (2.8)	
**Admission status**				
Heart rate	74.02 ± 19.05	68.90 ± 20.54	71.91 ± 19.18	0.0006
Systolic pressure	119.33 ± 26.69	120.69 ± 31.40	120.40 ± 27.10	0.6687
LVEDD (mm)	49.17 ± 6.66	49.45 ± 5.08	48.08 ± 7.26	0.0006
LVEF (%)	52.91 ± 9.60	54.27 ± 8.85	55.15 ± 8.64	<0.0001
Arrhythmia	92 (12.6)	53 (18.9)	140 (14.4)	0.0428
Ventricular flutter/fibrillation	10 (1.4)	11 (3.9)	30 (3.1)	0.0200
Atrial flutter/fibrillation	8 (1.1)	5 (1.8)	12 (1.2)	0.6971
Atrial-ventricular block	51 (7.0)	26 (9.3)	80 (8.2)	0.4207
Other	22 (3.0)	11 (3.9)	18 (1.8)	0.1046
Killip				<0.0001
I	480 (65.5)	210 (74.7)	774 (79.5)	
II	129 (17.6)	43 (15.3)	124 (12.7)	
III	46 (6.3)	6 (2.1)	13 (1.3)	
IV	78 (10.6)	22 (7.8)	63 (6.5)	
**TIMI**				<0.0001
0	NA	278 (98.9)	776 (79.7)	
I	NA	1 (0.4)	93 (9.5)	
II	NA	1 (0.4)	34 (3.5)	
III	NA	1 (0.4)	71 (7.3)	
Cardiogenic shock	72 (9.8)	29 (10.3)	60 (6.2)	0.0074
Cardiac arrest	17 (2.3)	12 (4.3)	10 (1.0)	0.0029
Defibrillation	5 (0.7)	11 (3.9)	26 (2.7)	0.0006
CPR	8 (1.1)	7 (2.5)	9 (0.9)	0.1518
Temporary pacemaker	3 (0.4)	1 (0.4)	22 (2.3)	0.0007
IABP	1 (0.1)	0 (0.0)	12 (1.2)	0.0023
GRACE risk score	163.00 ± 39.54	152.64 ± 38.19	151.34 ± 33.84	<0.0001
**Procedural outcomes**				
Success rate of thrombolysis	NA	242 (86.1)	NA	
Rescue PCI after thrombolysis	NA	6 (2.1)	NA	
TIMI after PCI				<0.0001
0	NA	0 (0)	8 (0.8)	
I	NA	1 (16.7)	15 (1.5)	
II	NA	0 (0)	19 (2.0)	
III	NA	5 (83.3)	932 (95.7)	
**In-hospital medications**				
Morphia	12 (1.6)	17 (6.0)	53 (5.4)	<0.0001
Atropine	16 (2.2)	10 (3.6)	19 (2.0)	0.3178
Epinephrine	17 (2.3)	3 (1.1)	16 (1.6)	0.3393
Dopamine	55 (7.5)	25 (8.9)	62 (6.4)	0.3205
Aspirin	685 (93.5)	270 (96.1)	270 (96.1)	0.0001
Clopidogrel/ticagrelor	678 (92.5)	267 (95.0)	943 (96.8)	0.0003
GP IIb/IIIa receptor antagonists	166 (22.6)	29 (10.3)	641 (65.8)	<0.0001
Heparin	644 (87.9)	264 (94.0)	881 (90.5)	0.0094
Statins	685 (93.5)	271 (96.4)	935 (96.0)	0.0324
β receptor blocker	391 (53.3)	135 (48.0)	619 (63.6)	<0.0001
ACEI/ARB	306 (41.7)	125 (44.5)	459 (47.1)	0.0858
**Admission time**				
CCU days	4.81 ±4.82	4.59 ±3.91	4.14 ±3.41	0.0028
In-hospital days	11.78 ±7.84	10.93 ±6.98	9.88 ±4.79	<0.0001
**Discharge medications**				
Aspirin	564 (76.9)	245 (87.2)	821 (84.3)	<0.0001
Clopidogrel/ticagrelor	560 (76.4)	238 (84.7)	786 (80.7)	0.0067
Nitrate	325 (44.3)	159 (56.6)	421 (43.2)	0.0003
Calcium channel blocker	43 (5.9)	15 (5.3)	61 (6.3)	0.8325
Statins	550 (75.0)	246 (87.5)	793 (81.4)	<0.0001
β receptor blocker	334 (45.6)	126 (44.8)	538 (55.2)	0.0001
ACEI/ARB	282 (38.5)	117 (41.6)	418 (42.9)	0.1773

*ACEI/ARB, angiotensin-converting enzyme inhibitor/angiotensin receptor blocker; BMI, body mass index; CABG, coronary artery bypass graft; CCU, intensive care unit; COPD, chronic obstructive pulmonary disease; CPR, cardio-pulmonary resuscitation; IABP, intra-aortic balloon pump; LVEDD, left ventricular end-diastolic dimension; LVEF, left ventricular ejection fractions; MI, myocardial infarction; PCI, percutaneous coronary intervention; TIMI, thrombolysis in AMI*.

Comparison of in-hospital outcomes according to treatment strategies in patients with right ventricular infarction is detailed in Table 2. In summary, both thrombolysis (5.7 vs. 12.6%; OR: 0.42; 95% CI: 0.24–0.73; *P* = 0.0020; adjusted OR: 0.43; 95% CI: 0.22–0.85; *P* = 0.0155) and primary PCI (3.1 vs. 12.6%; OR: 0.22; 95% CI: 0.14–0.34; *P* < 0.0001; adjusted OR: 0.48; 95% CI: 0.27–0.87; *P* = 0.0151) were all associated with lower risk of all-cause mortality relative to no reperfusion therapy, even after multivariable adjustment. Moreover, primary PCI was associated with low risk of atrial-ventricular block (4.2 vs. 8.9%; OR: 0.45; 95% CI: 0.27–0.75; *P* = 0.0024; adjusted OR: 0.46; 95% CI: 0.23–0.91; *P* = 0.0257), cardiogenic shock (5.3 vs. 13.9%; OR: 0.35; 95% CI: 0.23–0.54; *P* < 0.0001; adjusted OR: 0.43; 95% CI: 0.23–0.83; *P* = 0.0115), and heart failure (8.5 vs. 23.5%; OR: 0.30; 95% CI: 0.21–0.43; *P* < 0.0001; adjusted OR: 0.35; 95% CI: 0.22–0.56; *P* < 0.0001) compared with thrombolysis both in unadjusted and multivariable analysis. Multivariable analysis indicated that thrombolysis (OR: 0.50; 95% CI: 0.26–0.97; *P* = 0.0412), primary PCI (OR: 0.34; 95% CI: 0.21–0.56; *P* < 0.0001) and provincial hospitals (OR: 0.53; 95% CI: 0.29–0.96; *P* = 0.0368) were independent predictors to decrease in-hospital all-cause mortality. Age, previous stroke, cardiogenic shock, and GRACE risk score were independent predictors to increase in-hospital all-cause mortality (Table 3).

**Table 2:** Comparison of in-hospital outcomes according to treatment strategies in patients with right ventricular infarction.

	***N* of patients with at least one event [cumulative incidence (%)]**	**Unadjusted OR (95 CI)**	***P*-value**	**Multivariable adjusted OR (95 CI)**	***P*-value**
**All-cause mortality**					
No reperfusion	92 (12.6)	Reference	Reference	Reference	Reference
Thrombolysis	16 (5.7)	0.42 (0.24, 0.73)	0.0020	0.43 (0.22, 0.85)	0.0155
Primary PCI^*^	30 (3.1)	0.22 (0.14, 0.34)	<0.0001	0.48 (0.27, 0.87)	0.0151
**Secondary outcome**					
No reperfusion	164 (22.4)	Reference	Reference	Reference	Reference
Thrombolysis	81 (28.8)	1.41 (1.03, 1.92)	0.0321	1.20 (0.80, 1.82)	0.3783
Primary PCI^*^	166 (17.0)	0.71 (0.56, 0.91)	0.0059	0.85 (0.60, 1.21)	0.3673
**AMI**					
No reperfusion	3 (0.4)	Reference	Reference	Reference	Reference
Thrombolysis	3 (1.1)	2.63 (0.53, 13.09)	0.2388	9.82 (0.95,101.81)	0.0556
Primary PCI	5 (0.5)	1.26 (0.30, 5.27)	0.7558	10.54 (0.98,113.52)	0.0521
**GABG**					
No reperfusion	0	Reference	Reference	Reference	Reference
Thrombolysis	0	NA	NA	NA	NA
Primary PCI	0	NA	NA	NA	NA
**Stroke**					
No reperfusion	10 (1.4)	Reference	Reference	Reference	Reference
Thrombolysis	3 (1.1)	0.78 (0.21, 2.86)	0.7076	2.26 (0.45, 11.42)	0.3229
Primary PCI	10 (1.0)	0.75 (0.31, 1.81)	0.5224	1.41 (0.38, 5.22)	0.6027
**Major bleeding**					
No reperfusion	29 (4.0)	Reference	Reference	Reference	Reference
Thrombolysis	11 (3.9)	0.99 (0.49, 2.01)	0.9756	0.70 (0.31, 1.58)	0.3877
Primary PCI	26 (2.7)	0.67 (0.39, 1.14)	0.1386	0.64 (0.32, 1.27)	0.2021
**Ventricular flutter/fibrillation**					
No reperfusion	32 (4.4)	Reference	Reference	Reference	Reference
Thrombolysis	26 (9.3)	2.23 (1.31, 3.82)	0.0034	1.35 (0.71, 2.56)	0.3635
Primary PCI	60 (6.2)	1.44 (0.93, 2.23)	0.1057	1.28 (0.73, 2.27)	0.3901
**Atrial flutter/fibrillation**					
No reperfusion	13 (1.8)	Reference	Reference	Reference	Reference
Thrombolysis	3 (1.1)	0.60 (0.17, 2.11)	0.4244	0.44 (0.10, 1.93)	0.2751
Primary PCI	6 (0.6)	0.34 (0.13, 0.91)	0.0311	0.62 (0.17, 2.33)	0.4805
**Sinus arrest/severe bradycardia**					
No reperfusion	19 (2.6)	Reference	Reference	Reference	Reference
Thrombolysis	13 (4.6)	1.82 (0.89, 3.74)	0.1018	2.01 (0.81, 4.98)	0.1294
Primary PCI^*^	15 (1.5)	0.59 (0.30, 1.16)	0.1278	1.26 (0.52, 3.08)	0.6116
**Atrial-Ventricular block**					
No reperfusion	38 (5.2)	Reference	Reference	Reference	Reference
Thrombolysis	25 (8.9)	1.79 (1.06, 3.02)	0.0302	1.89 (0.93, 3.84)	0.0806
Primary PCI^*^^#^	41 (4.2)	0.80 (0.51, 1.26)	0.3435	0.86 (0.44, 1.66)	0.6491
**Cardiogenic shock**					
No reperfusion	93 (12.7)	Reference	Reference	Reference	Reference
Thrombolysis	39 (13.9)	1.11 (0.74, 1.66)	0.6139	1.30 (0.70, 2.39)	0.4055
Primary PCI^*^^#^	52 (5.3)	0.39 (0.27, 0.55)	<0.0001	0.56 (0.32, 0.99)	0.0476
**Heart failure**					
No reperfusion	158 (21.6)	Reference	Reference	Reference	Reference
Thrombolysis	66 (23.5)	1.12 (0.81, 1.55)	0.5069	1.25 (0.80, 1.94)	0.3228
Primary PCI^*^^#^	83 (8.5)	0.34 (0.25, 0.45)	<0.0001	0.44 (0.29, 0.66)	<0.0001

* *Indicated that there were significant differences between thrombolysis and primary PCI in unadjusted analysis*;

# *Indicated that there were significant differences between thrombolysis and primary PCI in multivariable adjusted analysis*.

**Table 3:** Independent predictors of in-hospital and 2-year all-cause mortality.

	**In-hospital all-cause mortality**		**2-year all-cause mortality**	
	**OR (95% CI)**	***P***	**HR (95% CI)**	***P***
Thrombolysis vs. no reperfusion	0.50 (0.26, 0.97)	0.0412	0.65 (0.41, 1.01)	0.0580
Primary PCI vs. no reperfusion	0.34 (0.21, 0.56)	<0.0001	0.39 (0.28, 0.55)	<0.0001
Age	1.03 (1.00, 1.05)	0.0365	1.02 (1.01, 1.04)	0.0038
Male	0.79 (0.50, 1.26)	0.3231	0.83 (0.61, 1.14)	0.2497
**Symptoms onset to admission time, vs**. ** <3 h**				
3–6 h	1.32 (0.76, 2.29)	0.3193	1.13 (0.78, 1.64)	0.5192
7–12 h	1.69 (0.89, 3.22)	0.1098	1.65 (1.09, 2.50)	0.0181
13–24 h	1.56 (0.79, 3.08)	0.1977	1.15 (0.72, 1.85)	0.5542
2–7 d	1.10 (0.57, 2.15)	0.7769	0.93 (0.59, 1.47)	0.7638
Smoking	0.85 (0.54, 1.33)	0.4764	1.03 (0.77, 1.39)	0.8384
Heart failure	1.78 (0.63, 5.01)	0.2731	1.92 (1.03, 3.58)	0.0405
Previous MI	0.84 (0.39, 1.81)	0.6557	1.01 (0.64, 1.62)	0.9507
Hypertension	0.79 (0.53, 1.19)	0.2679	0.93 (0.71, 1.22)	0.6054
Diabetes	1.39 (0.87, 2.23)	0.1626	1.37 (1.01, 1.85)	0.0434
Hyperlipidemia	0.83 (0.34, 2.05)	0.6937	0.92 (0.53, 1.61)	0.7747
Previous stroke	1.74 (1.01, 2.99)	0.0445	1.73 (1.22, 2.45)	0.0021
**Killip vs. Killip I**				
II	0.73 (0.40, 1.33)	0.3070	1.21 (0.82, 1.77)	0.3358
III	1.36 (0.61, 3.03)	0.4479	1.28 (0.72, 2.28)	0.3923
IV	0.44 (0.15, 1.26)	0.1248	0.79 (0.37, 1.71)	0.5521
Cardiogenic shock	2.99 (1.43, 6.23)	0.0035	1.74 (1.01, 3.01)	0.0463
LVEF	0.98 (0.96, 1.01)	0.1628	0.98 (0.97, 1.00)	0.0144
ACEI/ARB	1.66 (0.76, 3.64)	0.2034	1.35 (0.79, 2.30)	0.2763
β blocker	0.91 (0.34, 2.41)	0.8504	1.12 (0.61, 2.05)	0.7086
GRACE risk score	1.02 (1.01, 1.03)	<0.0001	1.01 (1.01, 1.02)	0.0001
**Hospital level vs. county hospitals**				
Provincial hospitals	0.53 (0.29, 0.96)	0.0368	0.73 (0.48, 1.09)	0.1255
Municipal hospitals	0.88 (0.51, 1.51)	0.6435	0.95 (0.65, 1.37)	0.7673

*ACEI/ARB, angiotensin-converting enzyme inhibitor/angiotensin receptor blocker*.

Outcomes during 1-year follow-up in Table 4 also indicated that thrombolysis (10.0 vs. 18.3%; HR: 0.53; 95% CI: 0.35–0.80; *P* = 0.0023; adjusted HR: 0.57; 95% CI: 0.36–0.90; *P* = 0.0153) and primary PCI (4.9 vs. 18.3%; HR: 0.25; 95% CI: 0.18–0.35; *P* < 0.0001; adjusted HR: 0.41; 95% CI: 0.27–0.62; *P* < 0.0001) all could reduce the risk of all-cause mortality relative to no reperfusion therapy (Table 4). Primary PCI was also associated with lowest risk of MACCE relative to thrombolysis (16.0 vs. 28.9%; HR: 0.50; 95% CI: 0.38–0.65; *P* < 0.0001; adjusted HR: 0.66; 95% CI: 0.49–0.90; *P* = 0.0084) and no reperfusion therapy (16.0 vs. 29.0%; HR: 0.50; 95% CI: 0.41–0.62; *P* < 0.0001; adjusted HR: 0.66; 95% CI: 0.51–0.86; *P* = 0.0021), which was mainly driven by lower risk of revascularization with primary PCI compared with thrombolysis (7.8 vs. 15.2%; HR: 0.48; 95% CI: 0.33–0.71; *P* = 0.0002; adjusted HR: 0.57; 95% CI: 0.36–0.91; *P* = 0.0176). However, thrombolysis may increase the risk of revascularization compared with no reperfusion therapy (15.2 vs. 7.0%; HR: 2.39; 95% CI: 1.55–3.68; *P* < 0.0001; adjusted HR: 2.31; 95% CI: 1.35–3.95; *P* = 0.0022).

**Table 4:** Clinical outcomes at 1-year according to treatment strategies in patients with right ventricular infarction.

	***N* of patients with at least one event [cumulative incidence (%)]**	**Unadjusted HR (95 CI)**	***P*-value**	**Multivariable adjusted HR (95 CI)**	***P*-value**
**All-cause mortality**					
No reperfusion	131 (18.3)	Reference	Reference	Reference	Reference
Thrombolysis	28 (10.0)	0.53 (0.35, 0.80)	0.0023	0.57 (0.36, 0.90)	0.0153
Primary PCI^*^	47 (4.9)	0.25 (0.18, 0.35)	<0.0001	0.41 (0.27, 0.62)	<0.0001
**MACCE**					
No reperfusion	207 (29.0)	Reference	Reference	Reference	Reference
Thrombolysis	81 (28.9)	1.02 (0.79, 1.32)	0.8923	1.00 (0.74, 1.34)	0.9892
Primary PCI^*^^#^	153 (16.0)	0.50 (0.41, 0.62)	<0.0001	0.66 (0.51, 0.86)	0.0021
**AMI**					
No reperfusion	14 (2.3)	Reference	Reference	Reference	Reference
Thrombolysis	9 (3.5)	1.60 (0.69, 3.70)	0.2711	1.37 (0.51, 3.68)	0.5269
Primary PCI	17 (1.8)	0.84 (0.42, 1.71)	0.6379	0.71 (0.29, 1.74)	0.4499
**Revascularization**					
No reperfusion	43 (7.0)	Reference	Reference	Reference	Reference
Thrombolysis	39 (15.2)	2.39 (1.55, 3.68)	<0.0001	2.31 (1.35, 3.95)	0.0022
Primary PCI^*^^#^	72 (7.8)	1.15 (0.79, 1.68)	0.4696	1.33 (0.81, 2.17)	0.2594
**Stroke**					
No reperfusion	16 (2.6)	Reference	Reference	Reference	Reference
Thrombolysis	3 (1.2)	0.46 (0.13, 1.59)	0.2213	0.91 (0.22, 3.70)	0.8951
Primary PCI	17 (1.8)	0.74 (0.38, 1.47)	0.3932	1.24 (0.47, 3.24)	0.6639
**Major bleeding**					
No reperfusion	32 (5.2)	Reference	Reference	Reference	Reference
Thrombolysis	12 (4.6)	0.93 (0.48, 1.81)	0.8378	0.68 (0.32, 1.44)	0.3080
Primary PCI	35 (3.8)	0.77 (0.47, 1.24)	0.2748	0.70 (0.38, 1.28)	0.2489

*AMI, acute myocardial infarction; MACCE, major adverse cardiac and cerebrovascular event*.

* *Indicated that there were significant differences between thrombolysis and primary PCI in unadjusted analysis*;

# *Indicated that there were significant differences between thrombolysis and primary PCI in multivariable adjusted analysis*.

Two-year outcomes are detailed in Table 5. Both thrombolysis (11.0 vs. 20.9%; HR: 0.51; 95% CI: 0.34–0.75; *P* = 0.0007; adjusted HR: 0.59; 95% CI: 0.38–0.92; *P* = 0.0189) and primary PCI (6.3 vs. 20.9%; HR: 0.28; 95% CI: 0.21–0.38; *P* < 0.0001; adjusted HR: 0.50; 95% CI: 0.34–0.73; *P* = 0.0003) were all associated with lower risk of all-cause mortality relative to no reperfusion therapy, even after multivariable adjustment. Furthermore, primary PCI was associated with lower risk of MACCE (19.1 vs. 33.3%; HR: 0.52; 95% CI: 0.43–0.64; *P* < 0.0001; adjusted HR: 0.72; 95% CI: 0.56–0.92; *P* = 0.0092) relative to no reperfusion therapy. However, thrombolysis increased the risk of revascularization (15.5 vs. 8.7%; HR: 1.97; 95% CI: 1.30–2.98; *P* = 0.0014; adjusted HR: 1.90; 95% CI: 1.15–3.16; *P* = 0.0124) compared with no reperfusion therapy.

**Table 5:** Clinical outcomes at 2-year according to treatment strategies in patients with right ventricular infarction.

	***N* of patients with at least one event [cumulative incidence (%)]**	**Unadjusted HR (95 CI)**	***P*-value**	**Multivariable adjusted HR (95 CI)**	***P*-value**
**All-cause mortality**					
No reperfusion	146 (20.9)	Reference	Reference	Reference	Reference
Thrombolysis	30 (11.0)	0.51 (0.34, 0.75)	0.0007	0.59 (0.38, 0.92)	0.0189
Primary PCI^*^	60 (6.3)	0.28 (0.21, 0.38)	<0.0001	0.50 (0.34, 0.73)	0.0003
**MACCE**					
No reperfusion	235 (33.3)	Reference	Reference	Reference	Reference
Thrombolysis	83 (30.2)	0.92 (0.71, 1.18)	0.4868	0.95 (0.71, 1.27)	0.7474
Primary PCI^*^	183 (19.1)	0.52 (0.43, 0.64)	<0.0001	0.72 (0.56, 0.92)	0.0092
**AMI**					
No reperfusion	20 (3.4)	Reference	Reference	Reference	Reference
Thrombolysis	9 (3.6)	1.11 (0.50, 2.43)	0.7996	1.01 (0.40, 2.55)	0.9768
Primary PCI	25 (2.7)	0.85 (0.47, 1.54)	0.5954	0.79 (0.37, 1.69)	0.5369
**Revascularization**					
No reperfusion	52 (8.7)	Reference	Reference	Reference	Reference
Thrombolysis	39 (15.5)	1.97 (1.30, 2.98)	0.0014	1.90 (1.15, 3.16)	0.0124
Primary PCI^*^	88 (9.6)	1.15 (0.82, 1.63)	0.4129	1.36 (0.87, 2.12)	0.1756
**Stroke**					
No reperfusion	18 (3.1)	Reference	Reference	Reference	Reference
Thrombolysis	3 (1.2)	0.41 (0.12, 1.38)	0.1494	0.74 (0.19, 2.86)	0.6578
Primary PCI	22 (2.4)	0.84 (0.45, 1.57)	0.5869	1.28 (0.55, 3.00)	0.5723
**Major bleeding**					
No reperfusion	33 (5.5)	Reference	Reference	Reference	Reference
Thrombolysis	12 (4.7)	0.90 (0.46, 1.74)	0.7553	0.66 (0.31, 1.41)	0.2860
Primary PCI	37 (4.0)	0.78 (0.49, 1.25)	0.3024	0.72 (0.40, 1.30)	0.2753

* *Indicated that there were significant differences between thrombolysis and primary PCI in unadjusted analysis*.

**Figure 2:**
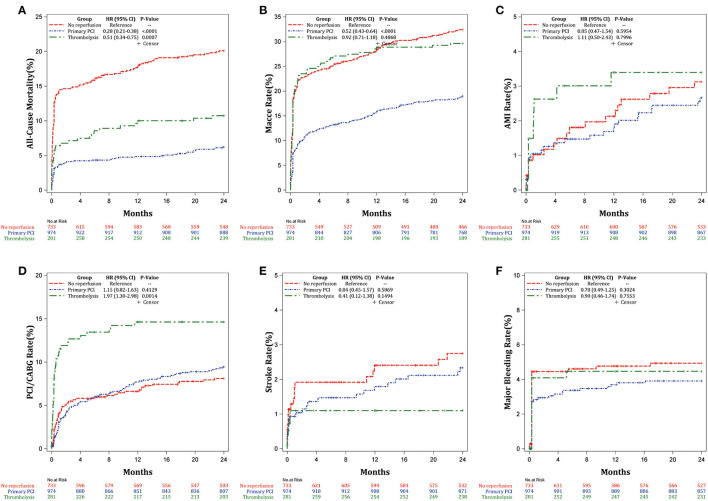
Kaplan-Meier estimates of the cumulative incidence of the outcomes after 2-years follow-up. **(A)** All-cause mortality, **(B)** MACCE, **(C)** AMI, **(D)** Revascularization, **(E)** Stroke, and **(F)** Major bleeding.

**Figure 3:**
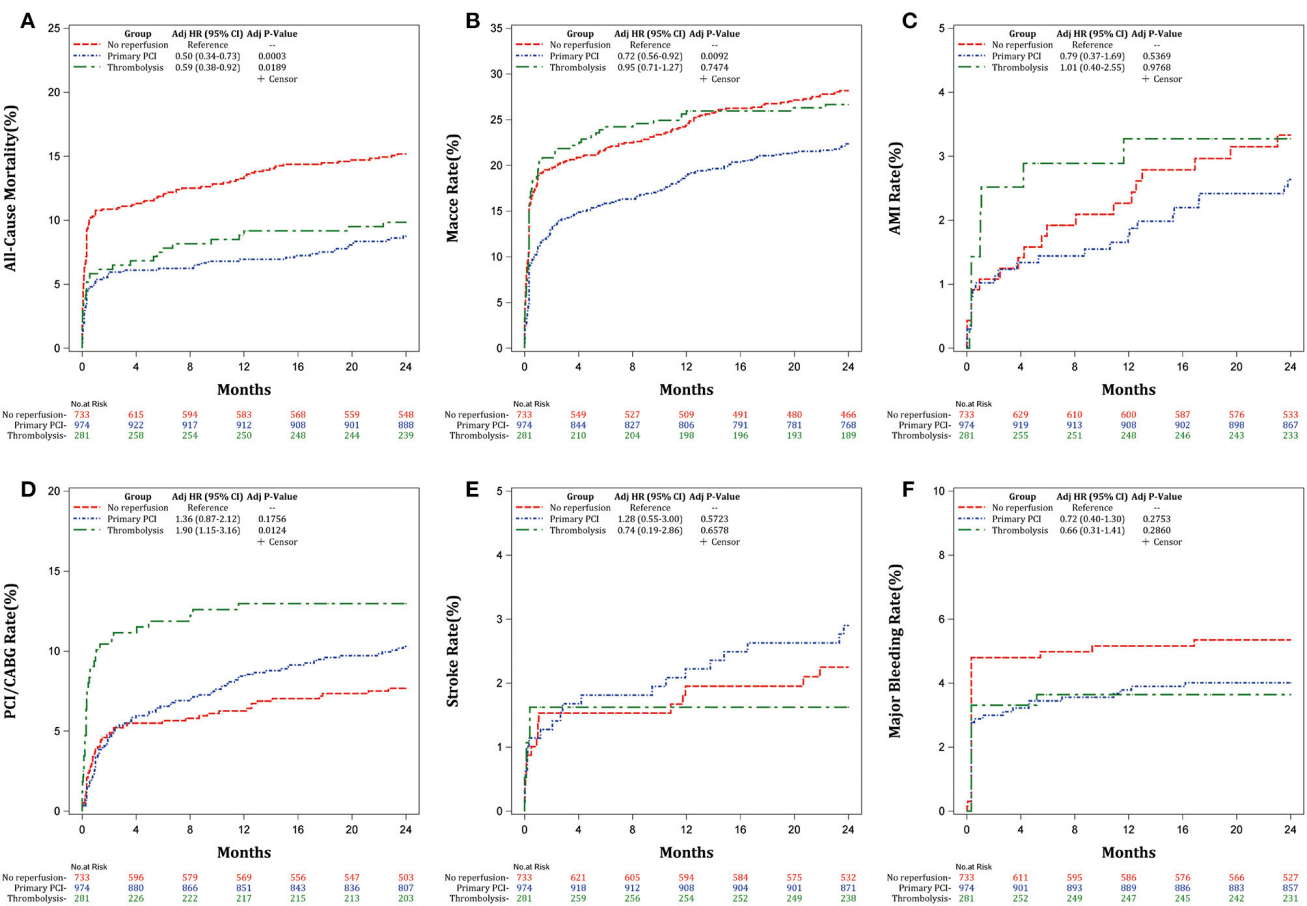
Adjusted Kaplan-Meier estimates of the cumulative incidence of the outcomes after 2-years follow-up. **(A)** All-cause mortality, **(B)** MACCE, **(C)** AMI, **(D)** Revascularization, **(E)** Stroke, and **(F)** Major bleeding.

Kaplan–Meier estimates of the cumulative incidence of outcomes after 2-year follow-up in Figure 2 also suggested that primary PCI and thrombolysis could reduce the risk of all-cause mortality (*P* < 0.0001; Figure 2A) relative to no reperfusion. Primary PCI was associated with lowest risk of MACCE (*P* < 0.0001; Figure 2B), whereas thrombolysis may increase the risk of revascularization (*P* = 0.0032; Figure 2D). There were no significant differences in AMI (Figure 2C), stroke (Figure 2E), and major bleeding (Figure 2F) among primary PCI, thrombolysis, and no reperfusion therapy groups. The abovementioned Kaplan-Meier estimates of outcomes remained consistent after multivariable adjustment (Figure 3).

Multivariable analysis indicated that primary PCI was an independent predictor to decrease 2-year (HR: 0.39; 95% CI: 0.28–0.55; *P* < 0.0001) all-cause mortality. Age, symptoms onset to admission time between 7 and 12 h, heart failure, diabetes, previous stroke, cardiogenic shock, and GRACE risk score were independent predictor to increase 2-year all-cause mortality (Table 3).

## Discussion

In the Chinese people-based registry, the main findings of our analysis can be summarized as follows: (1) nearly one-half patients with right ventricle infarction received primary PCI in China; (2) primary PCI and thrombolysis were all associated with lower risks of in-hospital and long-term all-cause mortality compared with no reperfusion therapy; (3) primary PCI could reduce the risks of in-hospital atrial-ventricular block, cardiogenic shock, heart failure, and long-term MACCE and revascularization, whereas thrombolysis may increase the risk of long-term revascularization; (4) primary PCI was an independent predictor to decrease both in-hospital (HR: 0.34; 95% CI: 0.21–0.56; *P* < 0.0001) and 2-year (HR: 0.39; 95% CI: 0.28–0.55; *P* < 0.0001) all-cause mortality.

Early randomized trials have confirmed that primary PCI is the standard treatment strategy for patients with STEMI when performed in a timely manner (14–17). The latest European Society of Cardiology (ESC) guideline also recommends that reperfusion therapy is indicated in all STEMI patients with symptoms of ischemia of ≤ 12 h, and primary PCI is over thrombolysis within indicated timeframes (18). However, the population was largely based on patients with left ventricular infarction, and the right and left ventricles differ markedly in their anatomy, mechanics, loading conditions, and metabolism. Therefore, they have strikingly different oxygen supply and demand characteristics (19), and thus manifest disparate responses to ischemic insults. Right ventricle oxygen demand is lower owing to lesser myocardial mass, preload, and afterload (19). Right ventricle perfusion is also more favorable because of the dual anatomic supply system from the left coronary branches. Furthermore, the right ventricle free wall is thinner, develops lower systolic intramyocardial pressure, and experiences less diastolic intracavitary pressure. Therefore, the lower coronary resistance favors acute collateral development to the right coronary artery (20), which makes right ventricular infarction often silent, with only 25% of patients developing clinically evident hemodynamic manifestations (21). However, there are limited and conflicting clinical outcomes on the effects of interventions designed to achieve reperfusion in right ventricular infarction. Some authors suggested that right ventricular function improves only after successful thrombolysis (4, 6, 7), whereas others reported recovery even in the absence of early recanalization (8). Meanwhile, there are scant data on the effects of primary PCI in patients with acute ischemic right ventricular dysfunction. Previous studies reported rapid hemodynamic improvement and an excellent clinical outcome after reperfusion in patients with right ventricular infarction who underwent primary angioplasty (7), whereas failure to restore flow to the major right ventricular branches was associated with lack of recovery of right ventricular performance and high in-hospital mortality (21, 22). Moreover, successful mechanical reperfusion also leads to superior late survival of patients with shock from predominant right ventricular infarction versus those with left ventricular shock (23), which highlights the importance of successful reperfusion in patients with right ventricular infarction. Our observations are consonant with the abovementioned studies, as indicated by the lower risks of in-hospital and long-term all-cause mortality with primary PCI and thrombolysis compared with no reperfusion therapy. Moreover, in our study, primary PCI could reduce the risks of in-hospital atrial-ventricular block, cardiogenic shock, heart failure, as well as long-term MACCE and revascularization.

Right ventricle infarction leads to an impaired contraction, which in turn leads to decreased ejection fraction of the right ventricle, decreased left ventricular filling, and hence a low cardiac output state and hypotension. This hypotension can progress to frank cardiogenic shock if left untreated or treated inadequately. As indicated in our study, the percent of cardiogenic shock was 8.1% in patients with right ventricular infarction, which was higher than that in our total STEMI population (3.9%) (24). Although the right ventricle may be resistant to infarction and usually recovers even after prolonged occlusion, yet a shorter time taken for reperfusion and complete revascularization of the affected vessels play an important role in the recovery of right ventricular function. Early revascularization leads to an immediate recovery of right ventricular function; conversely, late revascularization is associated with higher right ventricular dysfunction and complications (25). Previous study showed that complete reperfusion can improve right ventricular function and was independently associated with improved 30-day mortality (OR: 0.4; 95% CI: 0.1–1.05; *P* = 0.06) (1). Moreover, as shown by Bowers et al. early and complete reperfusion of the right coronary artery by angioplasty resulted in the dramatic recovery of right ventricular performance and an excellent clinical outcome, whereas unsuccessful reperfusion was associated with impaired recovery of right ventricular function and high all-cause mortality (4). Consistent with previous studies, we also found that reperfusion strategy was associated with lower risk of in-hospital and long-term all-cause mortality, and the risk of cardiogenic shock was lowest in patients undergoing primary PCI.

The higher incidence of heart block in association with right ventricular infarction is probably due to the involvement of the region of the atrioventricular node which is supplied by the right coronary artery. Ventricular tachycardia/ventricular fibrillation may develop either during acute occlusion, abruptly with reperfusion, or in a later phase. In our study, primary PCI dramatically reduces the incidence of malignant ventricular arrhythmias and heart blocks, presumably through improvement in right ventricular function, which lessens late ventricular arrhythmias and heart block (4, 26). Moreover, increased risks for bradyarrhythmia and ventricular tachyarrhythmias contribute to increased risk of in-hospital morbidity and mortality (21, 27). Therefore, the worse prognosis in patients with right ventricular myocardial involvement may be related to the increased risk of atrioventricular block and life-threatening ventricular arrhythmias in these patients (28). However, primary PCI was associated with fewer electrical complications and improved survival in our analysis, which was consistent with published studies (4, 29, 30).

Previous studies examining the prognostic impact of right ventricular infarction are mostly treated by thrombolytic therapy (27, 28). Our included patients with right ventricular infarction were also treated by primary PCI, which represents the current recommended therapy for STEMI patients. With this data analysis, we obtained a more reliable estimate of risk in the contemporary primary PCI era. Previous studies suggested that PCI with stenting seemed to be the most effective technique and as soon as right ventricular infarction is suspected, prompt primary PCI should be proposed (1), especially in those with severe hemodynamic compromise (22). Our findings also confirmed the benefits of primary PCI in patients with right ventricular infarction. Because in-hospital and long-term outcomes were poorer with no reperfusion therapy in our analysis, aggressive acute treatment including primary PCI, fluid resuscitation, temporary pacing, and mechanical support if needed should be considered for patients with right ventricular infarction to improve prognosis. However, despite the better prognosis with primary PCI, yet just 49.0% patients with right ventricular infarction received primary PCI in our analysis, which should be upgraded in further studies.

## Limitations

It is important to consider the limitations pertinent to the methods of this study. First, the present study was observational in nature, and was therefore subject to selection bias and other limitations inherent to such a study design. Therefore, our findings need to be confirmed prospectively in a well-organized randomized trial. Second, the relatively small sample size, especially of patients with thrombolysis, may decrease the statistical power of our analysis. Third, our study lacked systematic data (31). These variables are important, as they might be used as predictors to explain differences in cardiac prognosis.

## Conclusions

In our prospective, nationwide, multicenter CAMI registry, we found that primary PCI and thrombolysis were all associated with lower risks of in-hospital and long-term all-cause mortality compared with no reperfusion therapy. Especially, primary PCI was associated with lower risks of in-hospital atrial-ventricular block, cardiogenic shock, heart failure, and long-term revascularization compared with thrombolysis. Therefore, timely reperfusion therapy, especially primary PCI may be considered as the default treatment strategy for patients with right ventricular infarction.

## Data Availability Statement

The raw data supporting the conclusions of this article will be made available by the authors, without undue reservation.

## Ethics Statement

The studies involving human participants were reviewed and approved by the Institutional Review Board Committee of Fuwai Hospital. The patients/participants provided their written informed consent to participate in this study.

## Author Contributions

YY and XG: conceptualization, resources, validation, and writing—review and editing. MH and GC: formal analysis, validation, visualization, writing—original draft, and writing—review and editing. HY, JY, HX, YWu, and YWa: investigation, methodology, and software. LS, SQ, FH, WL, and CJ: data curation and formal analysis. All authors contributed to the article and approved the submitted version.

## Funding

This work was supported by the CAMS Innovation Fund for Medical Sciences (CIFMS) (2016-I2M-1-009) and the Twelfth Five-Year Planning Project of the Scientific and Technological Department of China (2011BAI11B02).

## Conflict of Interest

The authors declare that the research was conducted in the absence of any commercial or financial relationships that could be construed as a potential conflict of interest.

## Publisher's Note

All claims expressed in this article are solely those of the authors and do not necessarily represent those of their affiliated organizations, or those of the publisher, the editors and the reviewers. Any product that may be evaluated in this article, or claim that may be made by its manufacturer, is not guaranteed or endorsed by the publisher.

## References

[1] Assali AR Teplitsky I Ben-Dor I Solodky A Brosh D Battler A. Prognostic importance of right ventricular infarction in an acute myocardial infarction cohort referred for contemporary percutaneous reperfusion therapy. Am Heart J. (2007) 153:231–7. 10.1016/j.ahj.2006.10.038 17239681

[2] Bates ER Califf RM Stack RS Aronson L George BS Candela RJ. Thrombolysis and angioplasty in myocardial infarction (TAMI-1) trial: influence of infarct location on arterial patency, left ventricular function and mortality. J Am Coll Cardiol. (1989) 13:12–8. 10.1016/0735-1097(89)90542-1 2521226

[3] Dell'Italia LJ Lembo NJ Starling MR Crawford MH Simmons RS Lasher JC. Hemodynamically important right ventricular infarction: follow-up evaluation of right ventricular systolic function at rest and during exercise with radionuclide ventriculography and respiratory gas exchange. Circulation. (1987) 75:996–1003. 10.1161/01.CIR.75.5.996 3568315

[4] Bowers TR O'Neill WW Grines C Pica MC Safian RD Goldstein JA. Effect of reperfusion on biventricular function and survival after right ventricular infarction. N Engl J Med. (1998) 338:933–40. 10.1056/NEJM199804023381401 9521980

[5] Neumann FJ Sousa-Uva M Ahlsson A Alfonso F Banning AP Benedetto U. 2018 ESC/EACTS guidelines on myocardial revascularization. EuroIntervention. (2019) 14:1435–534. 10.4244/EIJY19M01_01 30667361

[6] Zehender M Kasper W Kauder E Geibel A Schönthaler M Olschewski M. Eligibility for and benefit of thrombolytic therapy in inferior myocardial infarction: focus on the prognostic importance of right ventricular infarction. J Am Coll Cardiol. (1994) 24:362–9. 10.1016/0735-1097(94)90289-5 8034869

[7] Kinn JW Ajluni SC Samyn JG Bates ER Grines CL O'Neill W. Rapid hemodynamic improvement after reperfusion during right ventricular infarction. J Am Coll Cardiol. (1995) 26:1230–4. 10.1016/0735-1097(95)00311-8 7594036

[8] Verani MS Tortoledo FE Batty JW Raizner AE. Effect of coronary artery recanalization on right ventricular function in patients with acute myocardial infarction. J Am Coll Cardiol. (1985) 5:1029–35. 10.1016/S0735-1097(85)80002-4 2985675

[9] Xu H Li W Yang J Wiviott SD Sabatine MS Peterson ED. The China acute myocardial infarction (CAMI) registry: a national long-term registry-research-education integrated platform for exploring acute myocardial infarction in China. Am Heart J. (2016) 175:193–201.e3. 10.1016/j.ahj.2015.04.014 27179740

[10] Thygesen K Alpert JS Jaffe AS Simoons ML Chaitman BR White HD. Third universal definition of myocardial infarction. J Am Coll Cardiol. (2012) 60:1581–98. 10.1016/j.gheart.2012.08.001 22958960

[11] Noguchi M Sakakura K Akashi N Adachi Y Watanabe Y Taniguchi Y. The comparison of clinical outcomes between inferior ST-elevation myocardial infarction with right ventricular infarction versus without right ventricular infarction. Int Heart J. (2019) 60:560–8. 10.1536/ihj.18-515 31105155

[12] Goldstein JA. Acute right ventricular infarction. Cardiol Clin. (2012) 30:219–32. 10.1016/j.ccl.2012.03.002 22548813

[13] Wiviott SD Braunwald E McCabe CH Montalescot G Ruzyllo W Gottlieb S. Prasugrel versus clopidogrel in patients with acute coronary syndromes. N Engl J Med. (2007) 357:2001–15. 10.1056/NEJMoa0706482 17982182

[14] Grines CL Browne KF Marco J Rothbaum D Stone GW O'Keefe J. A comparison of immediate angioplasty with thrombolytic therapy for acute myocardial infarction. The primary angioplasty in myocardial infarction study group. N Engl J Med. (1993) 328:673–9. 10.1056/NEJM199303113281001 8433725

[15] Zijlstra F de Boer MJ Hoorntje JC Reiffers S Reiber JH Suryapranata H. A comparison of immediate coronary angioplasty with intravenous streptokinase in acute myocardial infarction. N Engl J Med. (1993) 328:680–4. 10.1056/NEJM199303113281002 8433726

[16] Keeley EC Boura JA Grines CL. Primary angioplasty versus intravenous thrombolytic therapy for acute myocardial infarction: a quantitative review of 23 randomised trials. Lancet. (2003) 361:13–20. 10.1016/S0140-6736(03)12113-7 12517460

[17] Widimsky P Budesinsky T Vorac D Groch L Zelizko M Aschermann M. Long distance transport for primary angioplasty vs immediate thrombolysis in acute myocardial infarction. Final results of the randomized national multicentre trial–PRAGUE-2. Eur Heart J. (2003) 24:94–104. 10.1016/S0195-668X(02)00468-2 12559941

[18] Ibanez B James S Agewall S Antunes MJ Bucciarelli-Ducci C Bueno H. 2017 ESC guidelines for the management of acute myocardial infarction in patients presenting with ST-segment elevation: the task force for the management of acute myocardial infarction in patients presenting with ST-segment elevation of the European society of cardiology (ESC). Eur Heart J. (2018) 39:119–77. 10.1093/eurheartj/ehx393 28886621

[19] Ohzono K Koyanagi S Urabe Y Harasawa Y Tomoike H Nakamura M. Transmural distribution of myocardial infarction: difference between the right and left ventricles in a canine model. Circ Res. (1986) 59:63–73. 10.1161/01.RES.59.1.63 3731411

[20] Shiraki H Yoshikawa T Anzai T Negishi K Takahashi T Asakura Y. Association between preinfarction angina and a lower risk of right ventricular infarction. N Engl J Med. (1998) 338:941–7. 10.1056/NEJM199804023381402 9521981

[21] Goldstein JA. Pathophysiology and management of right heart ischemia. J Am Coll Cardiol. (2002) 40:841–53. 10.1016/S0735-1097(02)02048-X 12225706

[22] Jacobs AK Leopold JA Bates E Mendes LA Sleeper LA White H. Cardiogenic shock caused by right ventricular infarction: a report from the SHOCK registry. J Am Coll Cardiol. (2003) 41:1273–9. 10.1016/S0735-1097(03)00120-7 12706920

[23] Brodie BR Stuckey TD Hansen C Bradshaw BH Downey WE Pulsipher MW. Comparison of late survival in patients with cardiogenic shock due to right ventricular infarction versus left ventricular pump failure following primary percutaneous coronary intervention for ST-elevation acute myocardial infarction. Am J Cardiol. (2007) 99:431–5. 10.1016/j.amjcard.2006.09.091 17293178

[24] Xu H Yang Y Wang C Yang J Li W Zhang X. Association of hospital-level differences in care with outcomes among patients with acute ST-segment elevation myocardial infarction in China. JAMA Netw Open. (2020) 3:e2021677. 10.1001/jamanetworkopen.2020.21677 33095249 PMC7584928

[25] Kidawa M Kasprzak JD Wierzchowski T Krzeminska-Pakula M. Right ventricular function suffers from reperfusion delay: tissue doppler study. Clin Cardiol. (2010) 33:E43–8. 10.1002/clc.20582 20127894 PMC6653538

[26] Ricci JM Dukkipati SR Pica MC Haines DE Goldstein JA. Malignant ventricular arrhythmias in patients with acute right ventricular infarction undergoing mechanical reperfusion. Am J Cardiol. (2009) 104:1678–83. 10.1016/j.amjcard.2009.07.047 19962474

[27] Mehta SR Eikelboom JW Natarajan MK Diaz R Yi C Gibbons RJ. Impact of right ventricular involvement on mortality and morbidity in patients with inferior myocardial infarction. J Am Coll Cardiol. (2001) 37:37–43. 10.1016/S0735-1097(00)01089-5 11153770

[28] Kinch JW Ryan TJ. Right ventricular infarction. N Engl J Med. (1994) 330:1211–7. 10.1056/NEJM199404283301707 8139631

[29] Hanzel GS Merhi WM O'Neill WW Goldstein JA. Impact of mechanical reperfusion on clinical outcome in elderly patients with right ventricular infarction. Coron Artery Dis. (2006) 17:517–21. 10.1097/00019501-200609000-00004 16905963

[30] Goldstein JA Lee DT Pica MC Dixon SR O'Neill WW. Patterns of coronary compromise leading to bradyarrhythmias and hypotension in inferior myocardial infarction. Coron Artery Dis. (2005) 16:265–74. 10.1097/00019501-200508000-00002 16000883

[31] Li J Li X Wang Q Hu S Wang Y Masoudi FA. ST-segment elevation myocardial infarction in China from 2001 to 2011 (the China PEACE-retrospective acute myocardial infarction study): a retrospective analysis of hospital data. Lancet. (2015) 385:441–51. 10.1016/S0140-6736(14)60921-1 24969506 PMC4415374

